# New insights into the application of pair distribution function studies to biogenic and synthetic hydroxyapatites

**DOI:** 10.1038/s41598-020-73200-2

**Published:** 2020-11-11

**Authors:** Emily L. Arnold, Dean S. Keeble, Charlene Greenwood, Keith D. Rogers

**Affiliations:** 1 Cranfield Forensic Institute, Cranfield University, Shrivenham, SN6 8LA UK; 2 Diamond Light Source, Didcot, OX11 0QX UK; 3 School of Chemical and Physical Sciences, Keele University, Keele, ST5 5BJ UK

**Keywords:** X-ray diffraction, Biomaterials

## Abstract

Biogenic and synthetic hydroxyapatites are confounding materials whose properties remain uncertain, even after years of study. Pair distribution function (PDF) analysis was applied to hydroxyapatites in the 1970’s and 1980’s, but this area of research has not taken full advantage of the relatively recent advances in synchrotron facilities. Here, synchrotron X-ray PDF analysis is compared to techniques commonly used to characterise hydroxyapatite (such as wide angle X-ray scattering, Fourier-transform infrared spectroscopy and thermogravimetric analysis) for a range of biogenic and synthetic hydroxyapatites with a wide range of carbonate substitution. Contributions to the pair distribution function from collagen, carbonate and finite crystallite size were examined through principal component analysis and comparison of PDFs. Noticeable contributions from collagen were observed in biogenic PDFs when compared to synthetic PDFs (namely r < 15 Å), consistent with simulated PDFs of collagen structures. Additionally, changes in local structure were observed for PDFs of synthetic hydroxyapatites with differing carbonate content, notably in features near 4 Å, 8 Å and 19 Å. Regression models were generated to predict carbonate substitution from peak position within the PDFs.

## Introduction

Bone is a complex composite material primarily made up of 60–70 wt% mineral and 20–30 wt% type I collagen^1^ with the remaining constituents (< 10 wt%) non-collagenous proteins and water^2^. Bone mineral is often prototyped as hydroxyapatite, Ca_10_(PO_4_)_6_(OH)_2_, but is accepted to be heavily structurally disordered by vacancies and substitutions (at both anion and cation sites). Perhaps the most significant biological substitution is CO_3_^2−^ which can replace both PO_4_^3−^ and OH^−^, termed B-type and A-type respectively. CO_3_^2−^ constitutes between 5 and 9 wt% in biogenic apatites^3^, and is often also present at the surface of the crystallites (labile CO_3_^2−^) without being incorporated structurally. Many techniques are employed to examine bone chemistry, which is known to undergo molecular change with bone diseases (such as osteoporosis^4,5^ and primary and metastatic bone cancers^6,7^) and ageing^8,9^. Increasing CO_3_^2−^ substitution increases dissolution rates^10,11^ potentially contributing to abnormal pathologies observed in disease, notably osteoporosis which shows higher CO_3_^2−^ substitution^4,12,13^. Because of the nanocrystalline nature of biogenic hydroxyapatite, it can be problematic to characterise these materials reliably. Throughout this research both biogenic and synthetic apatites will be treated as a material rather than a biological problem.

Pair distribution function (PDF) techniques are a form of total scattering analysis (in this case X-ray total scattering) which considers not only Bragg peaks, but also diffuse scattering. The PDF is a real-space analysis technique which allows local structures to be analysed (atom–atom pair distances from 1 to 50 Å for some experimental designs), making it ideally suitable for nano-crystalline materials, such as biogenic hydroxyapatite (HA). With increasing access to synchrotron sources (and thus increased range of momentum transfer Q, enabling significantly greater real-space resolution) this technique has seen a rapid growth in its utilisation. As such, detailed accounts of this technique and all its derivatives are readily available^14–16^.

The use of PDFs for analysis of HA (both biologically derived and synthetic) as well as other calcium phosphates with applications to biological materials, was predominant in the 1970’s and 1980’s with the principal aim to confirm or refute the presence of amorphous calcium phosphate (ACP).

PDFs were first applied to HA by Harper and Posner in 1970^17^ on synthetic HA and stable ACP measured using a Cu source (and a Q_max_ of 7.45 Å^−1^). This was in an effort to explain a noticeable reduction in diffractogram intensity when biogenic HA was compared to synthetic apatites with a comparable 002 coherence length (CL), supposedly due to the presence of 45 wt% stable ACP, a theory proposed by Harper and Posner in 1966^18^. In the following years, research continued focusing mainly on differentiating ACP from HA^19,20^, determining the effect of carbonate substitution and different preparation mediums^21^. From these it was concluded that a maximum of 10 wt% ACP was present in biological bone mineral. Further studies found no evidence of a stable ACP phase present in mature bone mineral, but did propose that ACP could potentially be a precursor to poorly crystalline HA found in biogenic samples^22^, as seen within ACP-like deposits in the blue crab hepatopancreas^23^. Subsequently, calcium deficient HA was analysed and compared to biogenic HA^24^.

Subsequent research was undertaken examining the ACP precursor theory using bones from chicks at a range of ages (from embryonic to 2 years)^25^. This study found that though there is potentially contribution from the organic component (collagen and non-collagenous proteins) of bone in peaks at 2.4 Å and from 3 to 6 Å, no support for this ACP theory could be found, even in the youngest embryonic bones. Further studies proposed a method to determine differences in the rate of damping of the PDF with r^26,27^ called the ‘Modulation Ratio’ (MR):

1 $$ MR = \frac{{\sqrt {\mathop \sum \nolimits_{r = 15}^{25} G\left( {r_{m} } \right)^{2} /m} }}{{\sqrt {\mathop \sum \nolimits_{r = 1}^{7} G\left( {r_{n} } \right)^{2} /n} }} $$

where *G*(*r*_*n*_) is the amplitude of the PDF at point *n* for the set of values {*r*_1_ = 1, *r*_2_ = 1 + δ, …, *r*_*n*_ = 7} (where δ is the PDF spacing), *n* is the total number of values summed, *G*(*r*_*m*_) is the amplitude of the PDF at point *m* for the set of values {*r*_1_ = 15, *r*_2_ = 15 + δ, …, *r*_*n*_ = 25}, and *m* is the total number of values summed. MR is a semi-quantitative measure of the local (generally < 10 Å) and intermediate (normally 20–60 Å) structural order of the material in question. The MR is a ratio of the root mean square (RMS) of a high-r section of the PDF to the RMS of a low-r section of the PDF.

Most of the previous studies were performed with laboratory Cu k_α_ or Mo k_α_ sources, thus limiting Q_max_ to about 8 Å^−1^ or 17 Å^−1^ respectively. Overall, it was found that while the absence of calcium decreased PDF amplitudes slightly, a greater disturbance in intermediate and long-range order was more evident with greater carbonate substitutions^24^.

More recently synchrotron beamlines dedicated to PDF measurement with increased momentum transfer (Q_max_ = 30 Å^−1^–40 Å^−1^), have become available holding the promise of higher resolution studies. Marisa et al. examined synthetic B-type carbonated HAs (CO_3_^2−^ for PO_3_^4−^ exchange) to determine the orientation of the carbonate ion at the phosphate site^28^. This demonstrated the use of PDFs to examine the effects of carbonate on the surrounding environment, as well as the use of PDFs to detect carbonate reliably enough for structure refinement through a ‘real-space Rietveld’ refinement procedure.

Currently the technique most often used to differentiate between A- and B-type CO_3_^2−^ is Fourier-transform infrared spectroscopy (FTIR)^29–31^ which exploit the lattice site degeneracy of the ν_2_CO_3_^2−^ absorption band. However, the significant overlapping of bands produces equivocal quantification of carbonate that is not mitigated by peak fitting algorithms.

Our research aims to collect PDF data from a series of synthetic samples (with a range of carbonate substitutions), and a range of biogenic samples. Wide angle X-ray scattering (WAXS), attenuated total reflectance Fourier-transform infrared spectroscopy (ATR-FTIR), differential scanning calorimetry (DSC), thermogravimetric analysis (TGA), and PDF studies were used to examine both biogenic and synthetic HAs. Analysis was focused on examining the contribution of collagen to the PDF of biogenic samples, as well as elucidating the effects of carbonate substitution on both synthetic and biogenic HA. To forward this research area within context biogenic materials, it is essential to understand the influence of collagen on PDFs. Only once the effects of collagen are understood can the effects of CO_3_^2−^ be examined within biogenic materials. In the literature only a small number of samples have been examined within each study (N < 8 for synthetic HA and N < 5 for biogenic HA); this study provides a larger sample size for both synthetic and biogenic HA. Additionally, this study examines biogenic HA with an increased momentum transfer (Q_max_ > 25 Å^−1^) previously unseen. This research aspires to demonstrate that PDF analysis can reveal systematic structural differences between HAs and also has the potential to add to the understanding of both synthetic and biogenic HAs as a whole.

## Results

**Figure 1:**
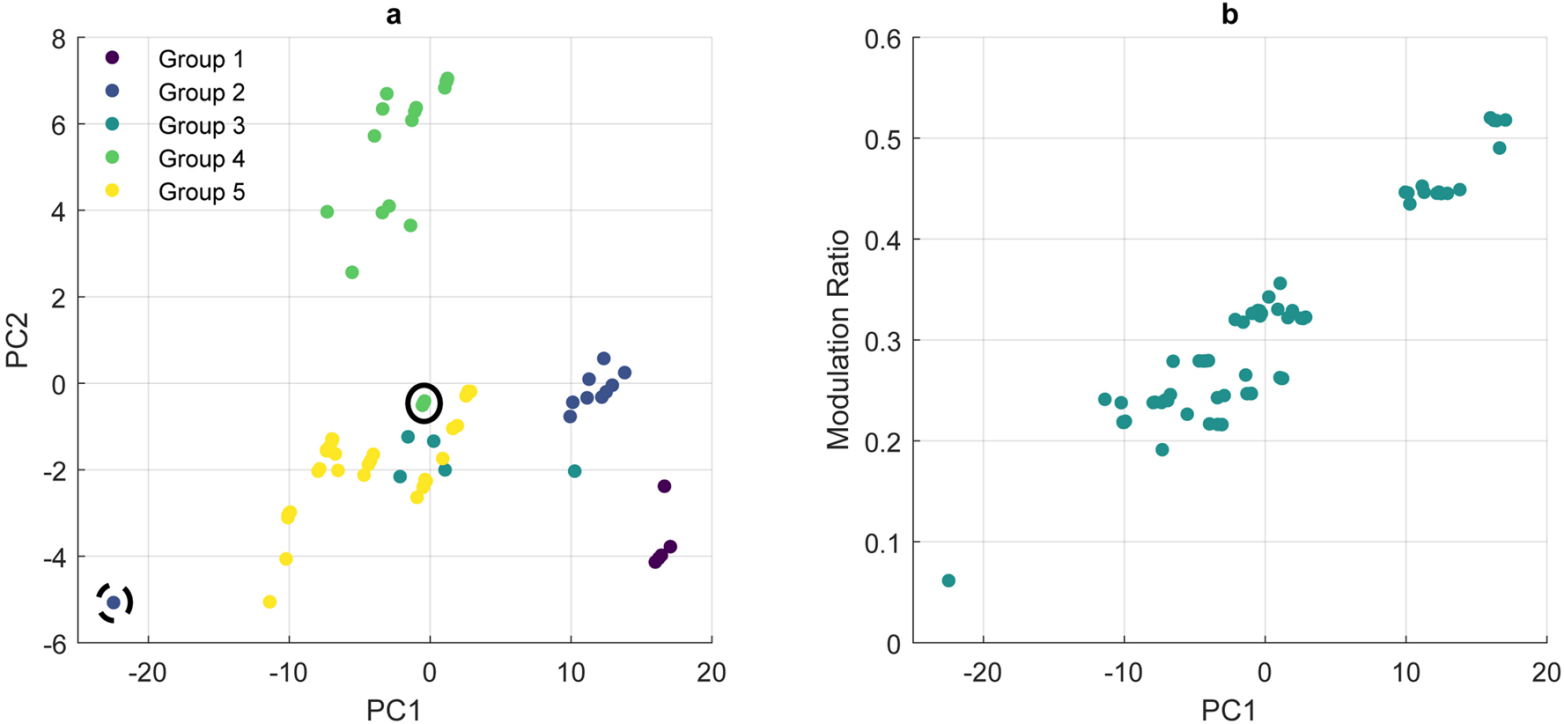
(**a**) PC 1 loading vs PC 2 loading (colour representative of sample groups, Rostrum is indicated by a solid circle, while ACP is indicated by a dashed circle), (**b**) the relationship between PC 1 and MR (R^2^ = 0.89, *p* ≪ 0.001).

For the unsupervised principal component analysis (PCA), the first five principal components (PC) were considered for further analysis (explaining 76.63%, 13.92%, 2.92%, 2.09% and 1.61% of variance with PC 1 to PC 5 respectively). Loadings for PC 1 and PC 2 were plotted against each other (Fig. 1a) to identify variation within the dataset. Results were plotted according to the sample group, and clusters can be seen, most prominently Group 4, made up of the majority of biogenic samples (only Rostrum is observed lower on the PC 2 axis, indicated by the solid circle). The ACP sample (from Group 2) is an obvious outlier and can be seen to have low values for both PC 1 and PC 2, indicated by the dashed circle. All repeated measurements were included and were observed to cluster more closely to measurements of the same sample than those of other samples, though some slight variation can be seen amongst both biogenic and synthetic samples, potentially due to slight beam damage. It can be seen in Fig. 1b that PC 1 correlates very well with MR (R^2^ = 0.89, *p* ≪ 0.001). PC 2, PC 3, PC 4 and PC 5 were also plotted in this manner and appeared to have little correlation with MR or CO_3_^2−^ wt%.

It is clear that ACP (the single outlier seen at ~ PC 1 = − 22.5 and PC 2 = − 5 in Fig. 1a) is significantly different from all other samples examined. As such, and as all subsequent analysis rely in some way on characterisation of the crystalline component of the samples, ACP is not included in further analyses.

### Collagen

For anisotropic crystallites (such as those with a rod-like or platy morphology), if the long axis dimension is much greater than the calculated PDF radius, then the PDF amplitude will be significantly altered by the radius (or thickness) of the crystallite in the smallest dimension^32^. In this case, the basal plane of HA represents the smallest dimension, best approximated through the 030 coherence length, CL, a measure of crystallite size and strain. Thus we have compared biogenic samples to synthetic samples with similar values of 030 CL, effectively mitigating effects of finite size on the PDF amplitude.

**Figure 2:**
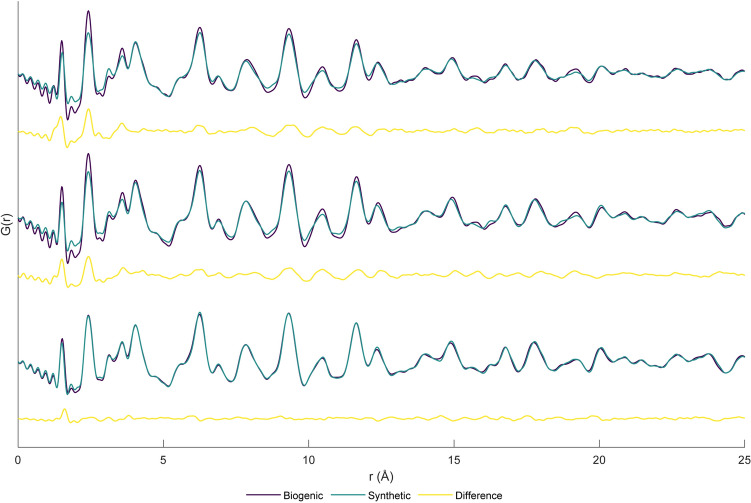
Comparison of biogenic and synthetic samples (and difference plots below) from bottom to top: mesoplodan rostrum (90.80 Ash%, 10.37 wt% CO_3_^2−^) and synthetic (7.52 wt% CO_3_^2−^), bovine (69.29 Ash%, 7.00 wt% CO_3_^2−^) and synthetic (8.12 wt% CO_3_^2−^), cervine antler (55.63 Ash%, 5.43 wt% CO_3_^2−^) and synthetic (5.83 wt% CO_3_^2−^).

This comparison is shown in Fig. 2, where three biogenic samples with a range of collagen content (ash% = 90.80%, 69.29% and 55.63% for mesoplodan rostrum, bovine, and cervine antler respectively) represent this relationship. The difference plots for both bovine and cervine antler comparisons are remarkably similar and possess a greater amplitude than for mesoplodan rostrum. These difference plots were compared with known collagen features. Higher orders of reflections from the D-period in collagen (well-established to be 67 nm) were not observed here.

Structures for ‘collagen-like peptides’ and ‘model collagen peptides’ were observed to have consistent peaks near 1.27 Å, 2.46 Å and 4.65 Å. The first, second and third peaks can be reliably attributed to first nearest neighbours (C–C, C–N, C–O and C=O), second nearest neighbours and neighbouring amino acid chains respectively. Synthetic collagen I structures were observed to have similar peaks near 1.20 Å, 2.52 Å and 4.66 Å directly comparable to those present within the collagen-like peptides simulated. Simulated PDFs of all structures examined can be seen in Supplementary Fig. S1 online. These particular peaks were also observed to be present within difference plots in Fig. 2. An additional peak near 3.54 Å is seen in both the bovine and cervine difference plots, which can be attributed to the distance from one amino acid to the next (e.g. N–N, O–O, C–C, etc.). While this peak cannot be easily observed within most simulations, it is present as a subpeak in the broad peak with a maximum near 4.65 Å.

### Effects of finite size

**Figure 3:**
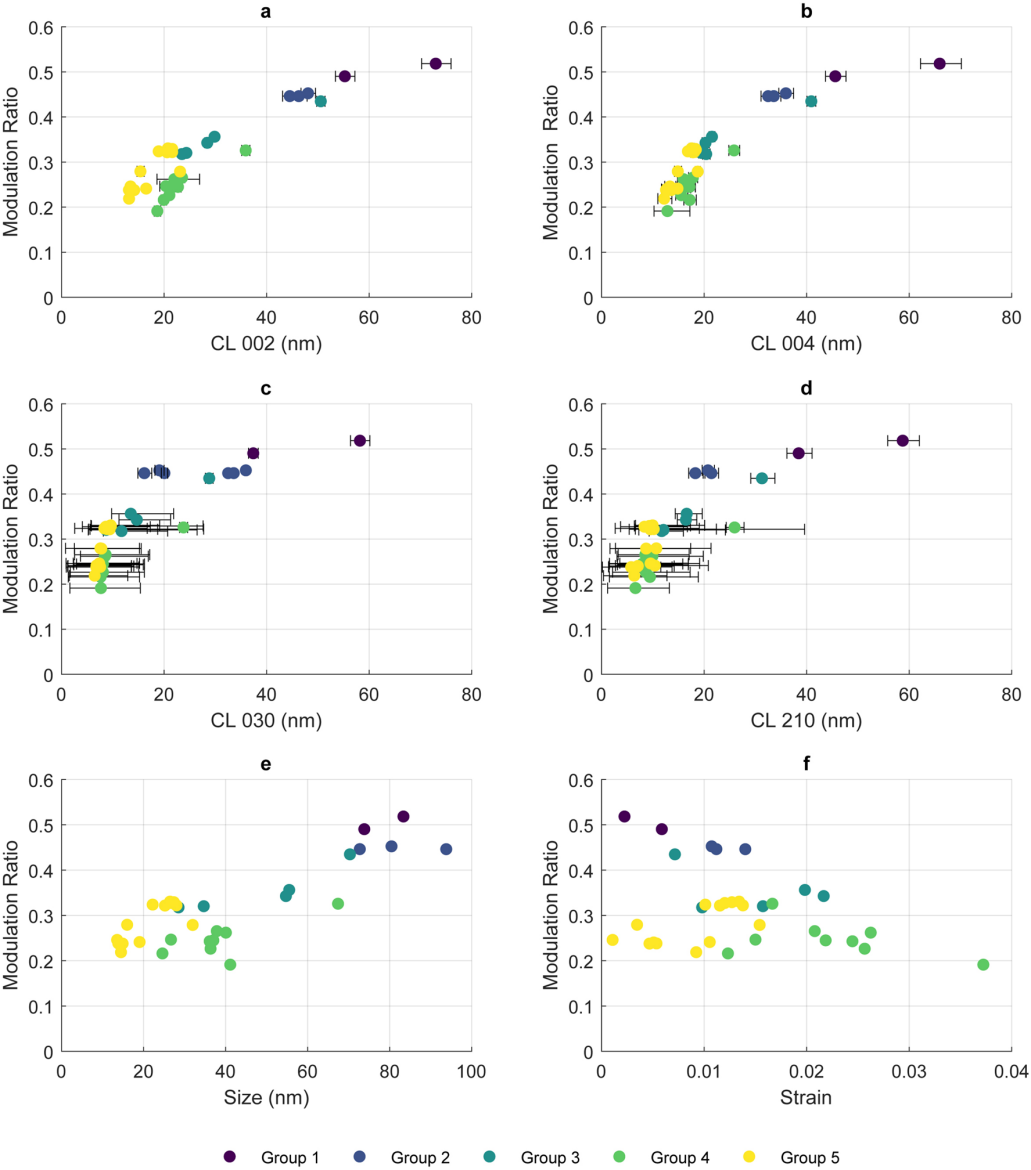
Relationship between MR and (**a**) 002 CL and MR, (**b**) 004 CL, (**c**) 030 CL, (**d**) 210 CL, (**e**) size (L) in <00ℓ> and (**f**) strain (ε) in <00ℓ>. Error bars represent peak fitting errors.

Coherence lengths derived from 002, 004, 030, and 210 Bragg maxima were plotted against MR (Fig. 3a–d respectively). Crystallite size (L) and strain (ε) along <00ℓ> were obtained from a Williamson-Hall analysis are presented within Fig. 3e and f respectively. Clear trends are seen between all CLs and MR, with higher variance observed for 002 than 004. Additionally, a correlation can be seen for 030 CL and 210 CL with MR. A weak relationship can be seen between crystallite size and MR, as is expected from the well-documented effects of finite size on the PDF amplitude. However, no discernible correlation is present for microstrain and MR.

**Figure 4:**
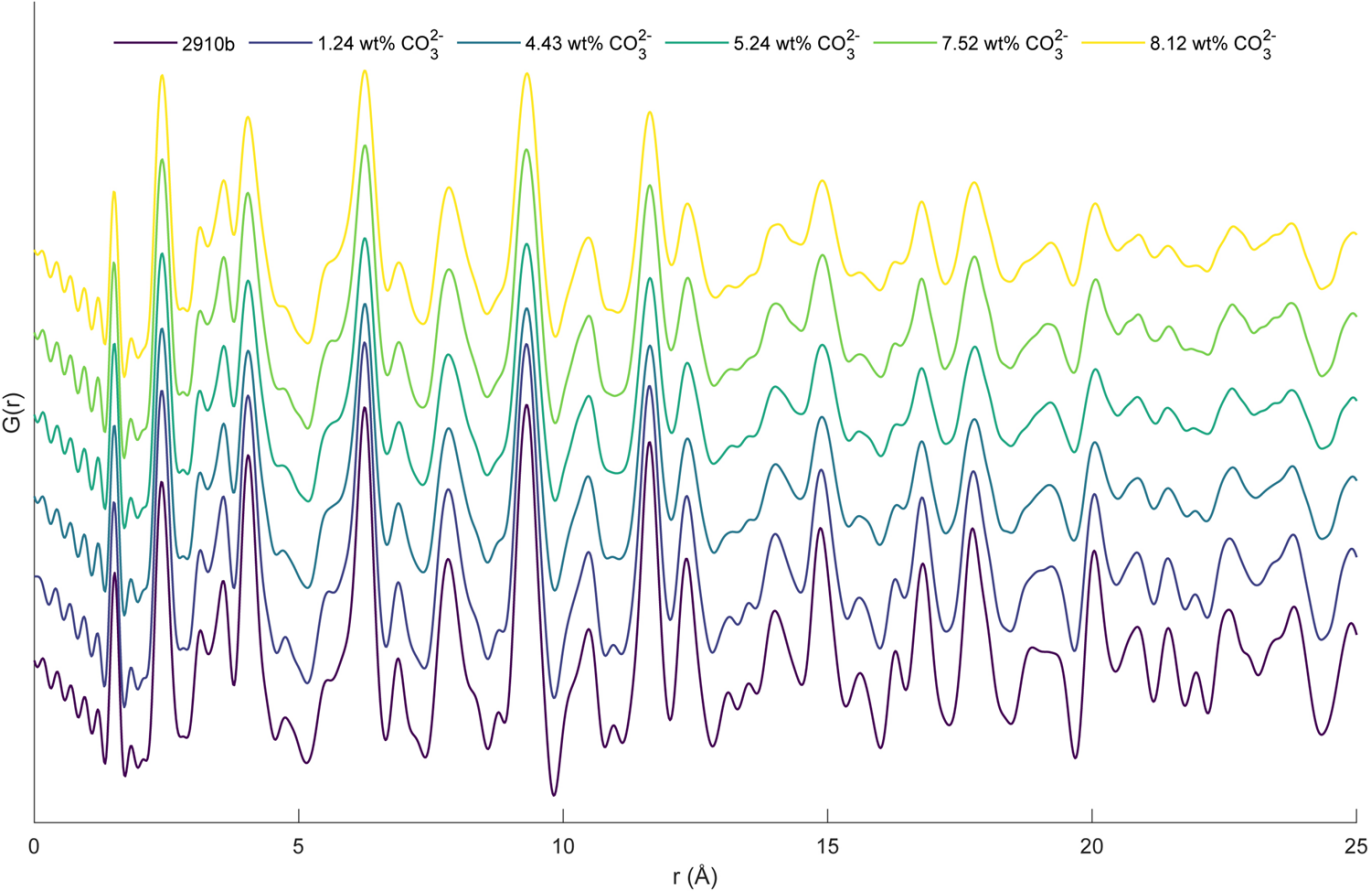
PDFs for synthetic samples with a range of carbonation from bottom to top: 2910b (below 0.40%), 1.24 wt% CO_3_^2−^, 4.43 wt% CO_3_^2−^, 5.24 wt% CO_3_^2−^, 7.52 wt% CO_3_^2−^, 8.12 wt% CO_3_^2−^.

**Figure 5:**
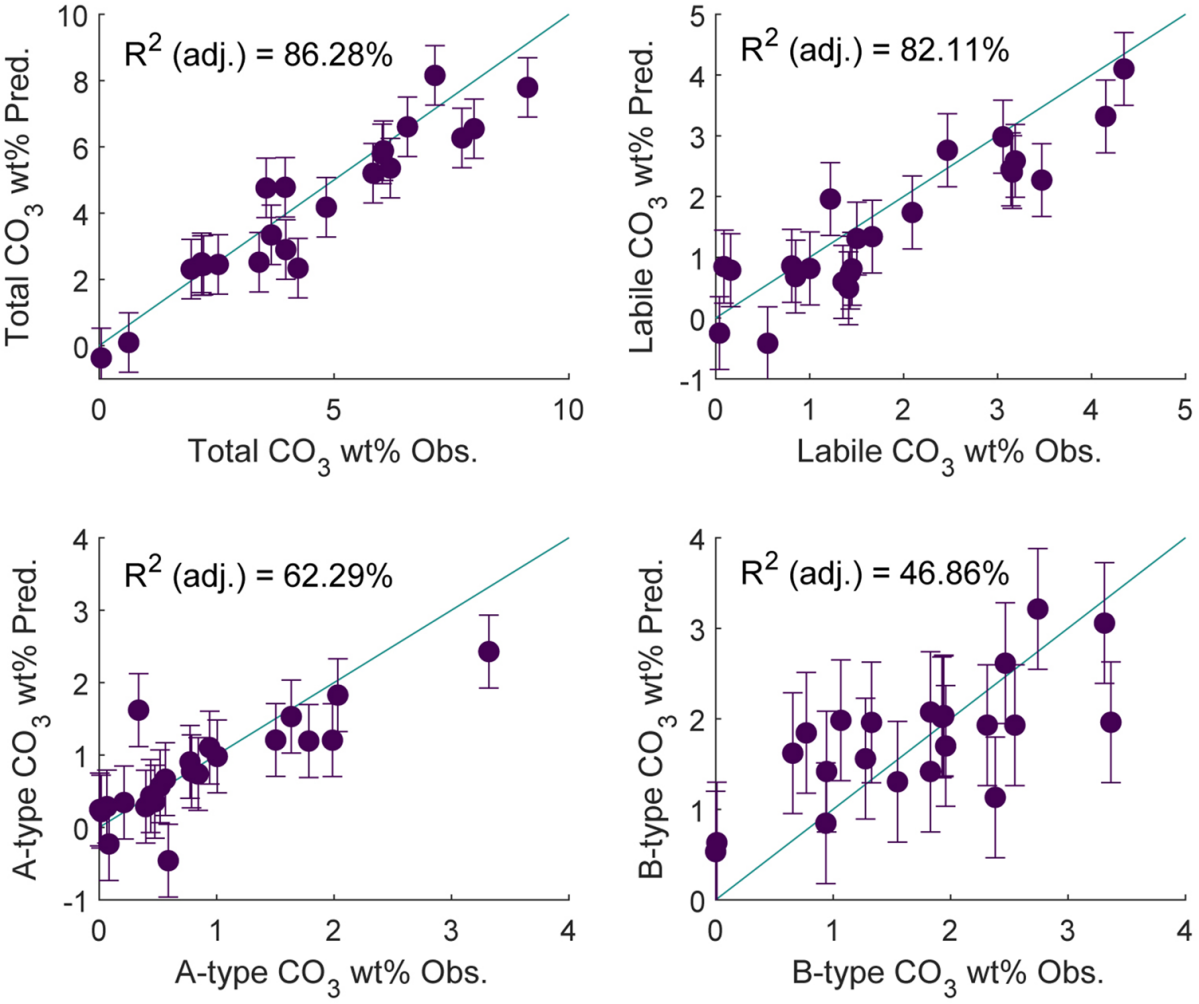
Observed vs predicted values for models generated through stepwise multiple regression for total CO_3_^2−^ wt%, labile CO_3_^2−^ wt%, A-type CO_3_^2−^ wt% and B-type CO_3_^2−^ wt%. Error bars represent the standard error of the regression (S).

A range of synthetic apatites with various amounts of carbonate substitution were measured and several corresponding data sets are shown in Fig. 4, (stacked from lowest to highest CO_3_^2−^ wt%). Features (such as that observed at r ~ 4 Å, ~ 8 Å and ~ 19 Å) can be seen to change significantly with CO_3_^2−^ content. Local maxima of ten peaks between 1 and 10 Å were measured (near ~ 1.51 Å, ~ 2.41 Å, ~ 3.13 Å, ~ 3.58 Å , ~ 4.04 Å, ~ 4.72 Å, ~ 6.25 Å, ~ 6.88 Å, ~ 7.84 Å and ~ 9.31 Å), and results were correlated to CO_3_^2−^ wt% (total, labile, A-type and B-type) for synthetic samples (peak used for this analysis can be seen in Supplementary Figure S2 online). A local maximum was not always present at peak 6 (near 4.72 Å), it is not included in further analyses. Total CO_3_^2−^ wt% was significantly correlated with seven of the nine peaks examined (*p* < 0.05), most strongly with the peaks near 2.41 Å, 3.58 Å and 7.84 Å (R^2^ = 0.52, 0.56 and 0.51 respectively). Similar significant relationships were seen between local maxima and A-type CO_3_^2−^ wt% (three peaks were significantly correlated, *p* < 0.5) and B-type CO_3_^2−^ wt% (four peaks were significantly correlated, *p* < 0.5). Stepwise regression was used to generate models which predict CO_3_^2−^ wt% with the use of peak positions with relative success (R^2^ (adj.) = 0.863, 0.781, 0.632 and 0.469 for total, labile, A-type and B-type CO_3_^2−^ wt% respectively). The observed and predicted results of these models can be seen in Fig. 5. All equations can be seen in Supplementary Table S1 and relationships between CO_3_^2−^ and all peaks used within each model can be seen in Supplementary Figure S3 online. Further, the relative damping changes are those expected from the relative decrease in 030 coherence length (58.20, 28.81, 13.51, 8.61, 14.68, 11.7 for 2910b, 1.24 wt% CO_3_^2−^, 4.43 wt% CO_3_^2−^, 5.24 wt% CO_3_^2−^, 7.52 wt% CO_3_^2−^, 8.12 wt% CO_3_^2−^ respectively).

## Discussion

Principal component analysis applied to the pair distribution function data indicates significant differences between all HA samples. In particular the damping rate of the PDFs shows significant variability with the characteristic modulation ratio, accounting for almost 77% of the inter-sample variation.

With regards to biologically derived samples, while very little variation can be seen in the difference plot for mesoplodan rostrum and synthetic HA, a similar pattern can be seen in the difference plots for both bovine and cervine antler. Both have above background intensity which persists to ~ 10–15 Å, and this is consistent with the width of a typical collagen type I triple helix (10–20 Å)^33^. From the structures examined, it is apparent that this low-r coherence is likely from intra-molecular bonds in amino acids (such as C=O, C–O, C–N, C–C). It is proposed that correlated motion between atoms on the same amino acid chain sharpens these peaks relative to peaks which arise from atoms which are in different amino acid chains, as is seen in Fig. 2. Collagen fibrils tend to be around 40 nm in diameter and 300 nm in length with a D-period of 67 nm^1^. The most likely orders to be present (those with the highest recorded intensity within the literature and where d < 50 Å) are n = 20 and 21, which would be present near 33.5 Å and 31.9 Å respectively^34^. As most studies of multiple orders of reflection for collagen study the preferentially oriented material^35,36^, whereas this study uses a powdered (and thus randomly oriented sample). As such, it is not expected that these higher order reflections would be observable, and within these samples they were not observed. A significant relationship (*p* = 0.005 and R^2^ = 0.70) is present between MR and the Ash% (an indicator of collagen content). With increasing collagen content, MR decreases indicating either a greater amplitude at low r (r = 1–7 Å) or more damped amplitude at high r (r = 15–25 Å), or both occurring simultaneously. From what can be seen in Fig. 2, when size effects are subtracted, an increase in several low r peaks is observed (namely r = 1.5 Å, 2.4 Å, 3.6 Å, 6.3 Å and 9.3 Å). Any previous studies have either used deproteinated biogenic HA^23^ or have simply ignored any effect collagen may have on the PDF^27^. However, it is apparent from this analysis of biogenic samples that collagen does have a significant effect on the PDF, as proposed by Glimcher et al.^25^.

While MR is significantly affected by collagen content it is also known to be affected by the scatterer finite size^32^. While MR appears to be a very useful tool for this analysis, it is important to acknowledge that PDF analysis is agnostic to crystallographic direction; crystallite anisotropy is not well represented. When measured using electron microscopy, biogenic HA is often found to be plate-like, with the greatest dimension along <00ℓ> of anywhere from 20 to 110 nm^37–39^ (dependant on species and preparation technique), a width of between 5 and 70 nm^37–39^, and a thickness between 1.5 and 3 nm^37,40^. The amplitude of the PDF can be calculated as if the crystallite is a nano-belt (according to Kodama^32^), with the thickness of the crystallite most likely to be represented by the 030 coherence length, as this most closely maps to the coherently diffracting domain across the short axis of the crystallite. However, characterising nano-crystalline hydroxyapatite through conventional profile deconvolution methods in the basal plane is problematic due to numerous overlapping reflections. The ability to observe local structure (< 30 Å, the thickness of biogenic HA) could provide an accurate measurement of the coherently diffracting domain along the shorter axis of HA crystallites.

When considering the relatively weak correlation between L in <00ℓ> and MR, this variation can most likely be explained as while <00ℓ> is the longest side of HA crystallites (and as such should have minimal effect on any damping), it is relatively well correlated with the crystallite width^37^ (in this case <0k0> , representing the short axis). While inhomogeneous strains (expected from substitutions such as CO_3_^2−^) would cause peak broadening at high-r (in turn causing reduced peak height) such strains would be sensitive to crystallographic direction. As with morphology, here microstrain has been measured along <00ℓ> due to the difficulty of separating size and strain contributions to peak broadening in other directions.

It is apparent that CO_3_^2−^ substitution is concomitant with particular features within the PDF (Fig. 4), most promisingly the feature near 19 Å and to a lesser extent at ~ 4 Å and ~ 8 Å. Also apparent is the systematic change in the PDF (observed through the shift in peak maxima) significantly correlated to the increase in CO_3_^2−^ substitution. Models generated by stepwise linear regression prove a mixed success (with strong R^2^ (adj) for total and labile CO_3_^2−^ wt% and less strong, though still promising, results for A-type and B-type CO_3_^2−^ wt%).

While the process of assigning peaks to the PDF of HA becomes increasingly difficult as r increases (particularly when a carbonated structure is considered) the first peak can be reliably assigned to an intramolecular P–O bond and the second peak to a convolution of a Ca–O atom pair and an intramolecular O–O atom pair. When carbonate is considered both an intramolecular C–O bond and an intramolecular O–O atom pair add to the first and second peaks respectively. When considering the trends seen within the peak positions with increasing CO_3_^2−^ (particularly peak 2 and peak 4 which show strong and significant relationships with total CO_3_^2−^ wt%; *p* < 0.001 for both peak 2 and peak 4 and R^2^ = 0.521 and 0.558 respectively) it is necessary to examine the atom pairs which contribute to each peak. Peak 2 (near 2.41 Å) is made up of O–Ca (2.41 Å) atom pairs with smaller contributions from O–O (2.45 Å). An increase in CO_3_^2−^ substitution will add an additional intramolecular O–O atom pair (with a distance of 2.12 Å). Further to this, comparison with the B-type substituted HA structure proposed by Iavanova et al*.*^41^ shows that consideration of the altered position of the O3 atom would increase the Ca–O atom pair distance from 2.41 to 2.49 Å. Even while the intramolecular O–O distance decreases, this shift would account for the overall increase in peak position (taking into account relative intensity of subpeaks due to differences in scattering power and number of atom pairs present). Peak 4 (near 3.58 Å) is primarily affected by P–Ca (at 3.61 Å) atom pairs, with small contributions from Ca–Ca (3.44 Å), O–O (3.46 Å) and P–O (3.71 Å). Again, comparison with Iavanova et al*.*^41^ shows that substitution in the ‘C2’ site would account for this shift; the P–Ca atom pair with a distance of 3.67 Å becomes a C–Ca pair with a distance of 3.76 Å. When compared to the differences which would be seen with a ‘C1’ substitution (this P–Ca distance of 3.67 Å becomes a C–Ca distance of 3.59 Å) the authors propose a preferential substitution into the C2 site over the C1 site for nanocrystalline B-type carbonated HA.

It is uncertain whether the damping observed with increased CO_3_^2−^ content arises solely from decreased size (well correlated with CO_3_^2−^ substitution in synthetic HAs^10,42^) or if it is complicated by irregularity of interatomic distances induced by CO_3_^2−^ substitution, as is proposed by Posner et al.^43^. While this work proposes that carbonate site distribution can be determined through the use of peak positions, this determination holds relatively large errors which may not be acceptable for more nuanced differences which may be present. This series shows promise for future structure fitting through the use of a real-space Rietveld-like refinement technique to more accurately determine CO_3_^2−^ site distribution, an achievement which is currently unavailable for nano-crystalline apatites.

With these differences clearly present (as can be seen from PCA, as well as through comparison of carbonated and biogenic samples) the use of PDFs for examination of both biogenic and synthetic HAs shows promise for structure refinement. As further quantitative work is carried forward, it should be noted that collagen significantly affects the PDF, and it is necessary to take into account collagen content throughout all stages of PDF processing and analysis. An understanding of CO_3_^2−^ can be achieved through study of PDFs of synthetic HA, and applied to PDFs of biogenic HA after collagen has been accounted for. The ability to understand structural CO_3_^2−^ could be very powerful within biomedical settings, providing further insight into physicochemical differences in healthy and diseased bone tissue.

## Materials and methods

### Materials

A wide range of synthetic and biologically derived apatites were used in this study. Synthetic sample sources included National Institute of Standards and Technology Standard Reference Material (NIST SRM) 2910b and 2910a (stoichiometric HA, subsequently referred to as Group 1), and three carbonated HA samples (0.5 wt%, 1.4 wt% and 2.3 wt% CO_3_^2−^) and an amorphous calcium phosphate sample sourced from Clarkson Chromatography Products Inc. (subsequently referred to as Group 2). Further, five carbonated HA samples were obtained from University of Exeter with 1.24 wt%, 4.43 wt%, 5.24 wt%, 7.52 wt% and 8.12 wt% CO_3_^2−^ (subsequently referred to as Group 3). An additional series of 14 synthetic HA samples (subsequently referred to as Group 5) was synthesised using the methods of Jarcho et al.^44^ and Merry et al.^45^ for stoichiometric and carbonated HA respectively. All synthesis was carried out at room temperature in an N_2_ atmosphere to exclude atmospheric sources of carbon.

Biogenic HA samples (Group 4) were harvested from 8 species, (9 individuals). These included femora from bovine *(Bos taurus)*, porcine *(Sus scrofa domesticus)*, cervine *(Cervus elaphus)*, galline *(Gallus gallus domesticus)*, canine *(Canis lupus familiaris)*, lapine *(Oryctolagus cuniculus)*, and cercopithecine *(Macaca mulatta)* as well as cervine antler and mesoplodan *(Mesoplodon densirostris)* rostrum. Biogenic specimens were obtained from several sources; the Animal Health Veterinary Laboratories Agency, local butchers, abattoirs, pet food stores and Bristol Veterinary College. All were stored at − 20 °C and the number of freeze/thaw cycles samples minimised as much as possible.

All samples were homogenised either mechanically (with a Retsch M2200 miller) or by hand using an agate mortar and pestle. Resultant powders were sieved using a 106 µm mesh to reduce preferred orientation effects and improve particle statistics. All samples are summarised in Table 1, including synthesis temperature for synthetic samples.

**Table 1:** Summary of sample groups, sample designations, and experimental PDF scans. All biogenic samples obtained from femurs unless otherwise stated. wt% refers to carbonate content.

Group no.	Synthetic/biogenic	Synthesis temperature	Total no of samples	Sample designation	No of PDF scans
1	Synthetic	105 °C	2	2910a	1
				2910b	4
2	Synthetic	25 °C	4	ACP, 1.4 wt%	1
				0.5 wt%, 2.3 wt%	4
3	Synthetic	80–90 °C	5	1.24 wt%, 4.43 wt%, 5.24 wt%, 7.52 wt%, 8.12 wt%	1
4	Biogenic	–	9	Cervine, galline, canine, lapine and cercopithecine	1
				bovine, porcine, cervine antler, mesoplodan rostrum	4
5	Synthetic	22 °C	14	2.18 wt%, 2.22 wt%, 3.40 wt%, 4.23 wt%, 5.56wt%, 5.83 wt%, 7.15 wt%, 9.12 wt%	1
				1.96 wt%, 2.54 wt%, 3.55 wt%, 6.03 wt%, 6.20 wt%, 7.98 wt%	4

### Wide angle X-ray scattering, WAXS

All WAXS data was collected using a PANalytical XPert PRO Multi-Purpose Diffractometer with Cu *K*_*α*_ radiation source. A PIXcel strip detector was used to collect data from 10° to 80° 2θ. Additional scans were performed over ranges 23°–28° 2θ and 55°–28° 2θ with a smaller step size and higher count time to improve the signal-to-noise ratio of both 002 and 004 Bragg peaks and enable subsequent Williamson-Hall analysis along <00ℓ> in a manner commonly used for HA^46,47^. Topas (Bruker, Version 4.1) was used to fit diffraction profiles using a full profile refinement procedure. In these refinements, ‘a’ axis, ‘c’ axis, and crystallite size in the hkℓ phase, and sample displacement variables were refined. Lattice parameters were calculated from additional measurements of all samples after spiking with an internal silicon standard (NIST SRM 640c). Coherence length (CL) was determined from 002, 030, 210, and 004 Bragg maxima using Scherrer’s Equtaion^46^, using a split-pseudo Voigt peak shape.

### ATR-FTIR

ATR-FTIR was used to determine carbonate levels in all samples. All ATR-FTIR data was collected using a ‘Bruker Alpha’ with a diamond ATR crystal, and a scan range from 4000 to 400 cm^−1^, with 64 averaged scans and 4 cm^−1^ resolution. Spectra were analysed using Spectrum (PerkinElmer) and background correction was performed before all subsequent analysis. Area and height were measured for the _ν1 ν3_PO_4_^3−^ peak and _ν2_CO_3_^2−^ peak. Each spectrum was deconvoluted until peak centres were apparent near 866 cm^−1^, 873 cm^−1^ and 878 cm^−1^ corresponding to labile, B-type and A-type CO_3_^2−^ respectively. PeakFit4 (Sigmaplot) was used to fit three peaks to all samples with measurable carbonate content (from the _ν2_CO_3_^2−^) after the spectra was cut to 910–840 cm^−1^ and baseline corrected. Baseline correction was applied linearly using start and end points of the _ν2_CO_3_^2−^ peak determined in initial analysis. Peak centres were fixed at values determined with deconvolution. A least square fitting technique was used to fit three Voigt peaks to each spectrum, varying several parameters including intensity, FWHM, and peak shape. Several parameters including peak intensity and integrated area were measured. This process was then completed three times for each spectrum and results were averaged. All samples from groups 1 and 2 were used to create a calibration curve, using the ratio of _ν2_CO_3_^2−^: _ν1 ν3_PO_4_^3−^ peak areas.

### DSC/TGA

DSC/TGA was used to determine ash weight (ash%) for biogenic samples. Biogenic samples were allowed to completely thaw (at room temperature for a minimum of 5 h) before analysis. 40 µL aluminium pans were filled with ~ 10 mg of powder. A hole was pierced in the lid of each pan before heating, and an empty aluminium crucible was used as a reference. The same measurement was performed with an empty aluminium crucible, which was used as a background. A Mettler Toledo TGA/DSC3 + (Indium calibrated) was used for all analysis. Temperatures interrogated included a range from 30 to 550 °C with a heating rate of 10 °C/min, with temperature sustained at 550 °C for 10 min at the end of the heating regime. Air flow rate was kept at a constant 50 mL/min, allowing combustion of organic material. STAR^e^ Thermal Analysis Software (Mettler Toledo, V 16.00) was used for analysis of both DSC and TGA curves. All curves were normalised to sample weight and background was subtracted. Ash weight % (ash%) was calculated for all samples by measuring the percentage weight loss from 50 to 550 °C (measured only after 10 min dwell). As structural carbonate is not driven from the HA lattice below 600 °C, it is not considered to contribute to weight loss measured by ash%^30,48^.

### Simulated PDFs

Collagen structures were found by searching the Protein Data Bank (PDB) using the search term “Collagen” as a macromolecular name. A total of 215 entries were returned and a total of 48 entries retained for this analysis. Any structures which included additional fragments or binding sites were excluded from this analysis. Out of these 48 entries, 44 contained the fractional atomic coordinates necessary to simulate a PDF from their structure. Out of these 44 structures, three originate from ‘synthetic collagen I’ while the remaining 41 originate from a ‘collagen-like peptide’ or ‘model collagen peptide.’ PDFGui^49^ was used to simulate PDFs of collagen. Simulations were performed with Q_max_ = ∞, and to r = 20 Å. Simulations were not considered beyond 20 Å as the collagen triple helix is known to have a diameter between 10 Å and 20 Å^33^ and its hierarchical structure (a complex of triple helices make up collagen fibres less than 50 Å, which in turn make up collagen fibrils) would dictate inter-molecular relationships not present within the PDB entries. All structures used can be seen in Supplementary Figure S1 (available online).

### Experimental PDFs

Experimental PDFs were acquired from the I15-1 beamline at Diamond Light Source, using 76.7 keV radiation (λ = 0.161669 Å). Two data sets were collected simultaneously, one used for total scattering analysis (Q-range = 0–38.86 Å^−1^) and the other used for WAXS (Q-range = 3.39–16.82 Å^−1^). DAWN^50^ was used to collapse the 2D area data to 1D line data. A total of 34 samples were measured (details of measurement can be seen in Table 1), with 13 of these samples being measured an additional 3 times to determine repeatability. PDFs were processed using GudrunX^51^ with a Q_min_ = 0.5 Å^−1^ and Q_max_ = 25.6 Å^−1^. Composition was calculated with the use of CO_3_^2−^ wt% for both synthetic and biogenic samples (assuming no additional substitution, Eq. (2) was charge balanced to determine HA composition), as well as ash% for biogenic samples (all loss of weight is assumed to be from combustion of collagen) where composition of type I collagen was taken from PDB entry 3HQV^52^. Density was calculated from HA composition and lattice parameters determined by WAXS.

2 $$ Ca_{{\left( {10 - x} \right)}} \left( {PO_{4} } \right)_{{\left( {6 - 2x} \right)}} \left( {CO_{3} } \right)_{2x} \left( {OH} \right)_{{\left( {2 - y} \right)}} \left( {CO_{3} } \right)_{y} $$

Matlab R2020a (Mathworks) was used to calculate MR for all PDFs using Eq. (1), as well as for principal component analysis (PCA) performed on all experimental PDFs. Each of the 73 PDFs were used as a single observation with 2501 variables. Singular value decomposition was used for analysis. Local maxima of peaks below 10 Å within PDFs were found using the findpeaks function within Matlab 2020a. Minitab17 was used to perform multiple regression with stepwise selection of terms for synthetic samples using peak positions to predict total CO_3_^2−^, labile CO_3_^2−^, A-type CO_3_^2−^ and B-type CO_3_^2−^.

## Supplementary information

Supplementary Information.
