# Supplementary material for: New insights into the application of pair distribution function studies to biogenic and synthetic hydroxyapatites
Source: Sci Rep. 2020 Nov 11;10:19597. doi: 10.1038/s41598-020-73200-2 (PMC7659341; doi:10.1038/s41598-020-73200-2)
Supplement: Supplementary file 1 — Supplementary Information. [file 41598_2020_73200_MOESM1_ESM.pdf]

New Insights into the Application of Pair Distribution Function Studies to Biogenic and Synthetic Hydroxyapatites

Emily L. Arnold, Dean S. Keeble, Charlene Greenwood, Keith D. Rogers

Supplementary Information

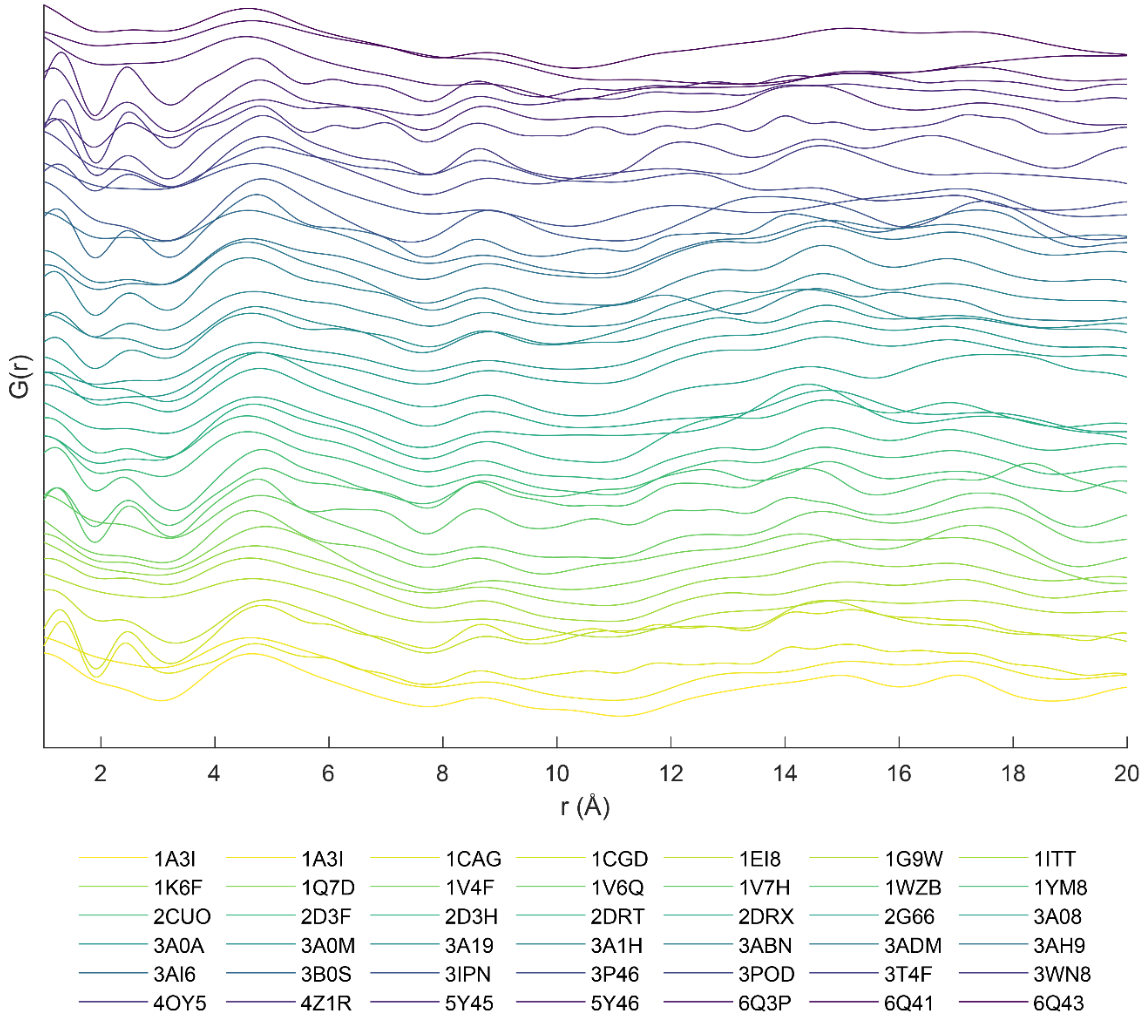

Supplementary Figure S1: All simulations of 'collagen-like peptides,' 'model collagen peptides' and 'synthetic collagen I' triple helix structures (normalised and offset from bottom to top): 1A3I[1], 1A3J[1], 1CAG[2], 1CGD[3], 1EI8[4], 1G9W[5], 1ITT[6], 1K6F[7],1Q7D[8], 1V4F[9], 1V6Q[9], 1V7H[9], 1WZB[10], 1YM8[11], 2CUO[12], 2D3F[13], 2D3H[13], 2DRX[14], 2G66[15], 3A08[16], 3A0A[17], 3A0M[18], 3A19[16], 3A1H[18], 3ABN[19], 3ADM[18], 3AH9[20], 3AI6[21], 3B0S[22], 3IPN[23], 3P46[24], 3POD[25], 3T4F[26], 3WN8[27], 4OY5[28], 4Z1R[29], 5Y45[30], 5Y46[30], 6Q3P[31], 6Q41[31] AND 6Q43[31]. All structures were simulated under the same conditions (no Q_{max} correction, r = 1 Å – 50 Å) and no rigid body or thermal motion corrections were applied. 6Q3P, 6Q41 and 6Q43 originate from 'synthetic collagen I' structures while all others originate from 'collagen-like peptide' and 'model collagen peptide' structures.

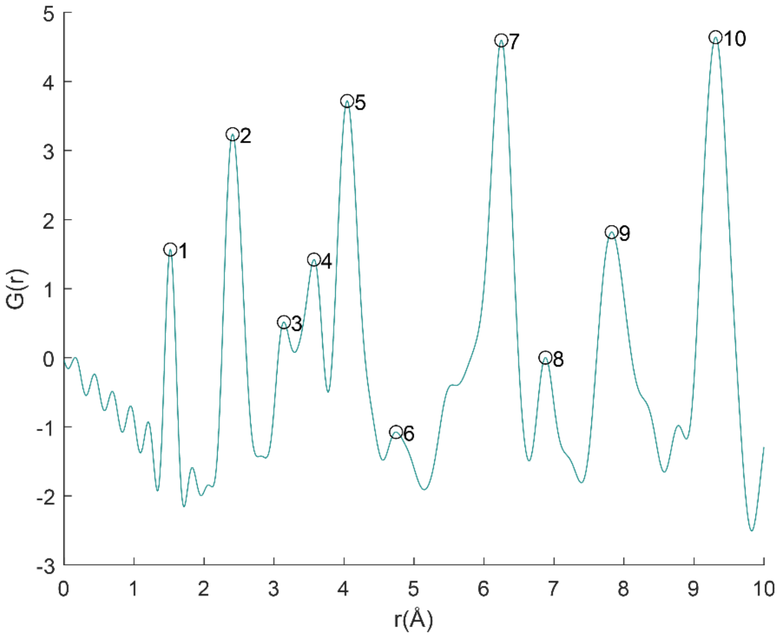

Supplementary Figure S2: Peaks used for stepwise multiple regression to predict carbonate wt%. Peaks were measured near 1.51 Å, 2.41 Å, 3.13 Å, 3.58 Å, 4.04 Å, 4.72 Å, 6.25 Å, 6.88 Å, 7.84 Å and 9.31 Å.

Supplementary Table S1: Results of stepwise regression models. Input for all models was nine of the ten peak maxima (the peak not included was peak 6, as it was not always present). Both alpha to enter and alpha to leave were set to 0.15. S (the standard error of the regression) and adjusted R^2 are given.

CO_3^{2-} wt%	Equation	S	R^2 (adj.)
Total	$-2244 - 112.3 \times Peak1 + 225 \times Peak4 + 148.3 \times Peak8 + 75.5 \times Peak9$	0.897	0.863
Labile	$-651 + 75.2 \times Peak4 + 88.3 \times Peak5 + 38.9 \times Peak8 + 60.3 \times Peak9$	0.600	0.781
A-Type	$-1522 + 107.2 \times Peak5 + 67.5 \times Peak7 + 97 \times Peak8$	0.503	0.623
B-Type	$-530 + 156.5 \times Peak2 + 43 \times Peak4$	0.667	0.469

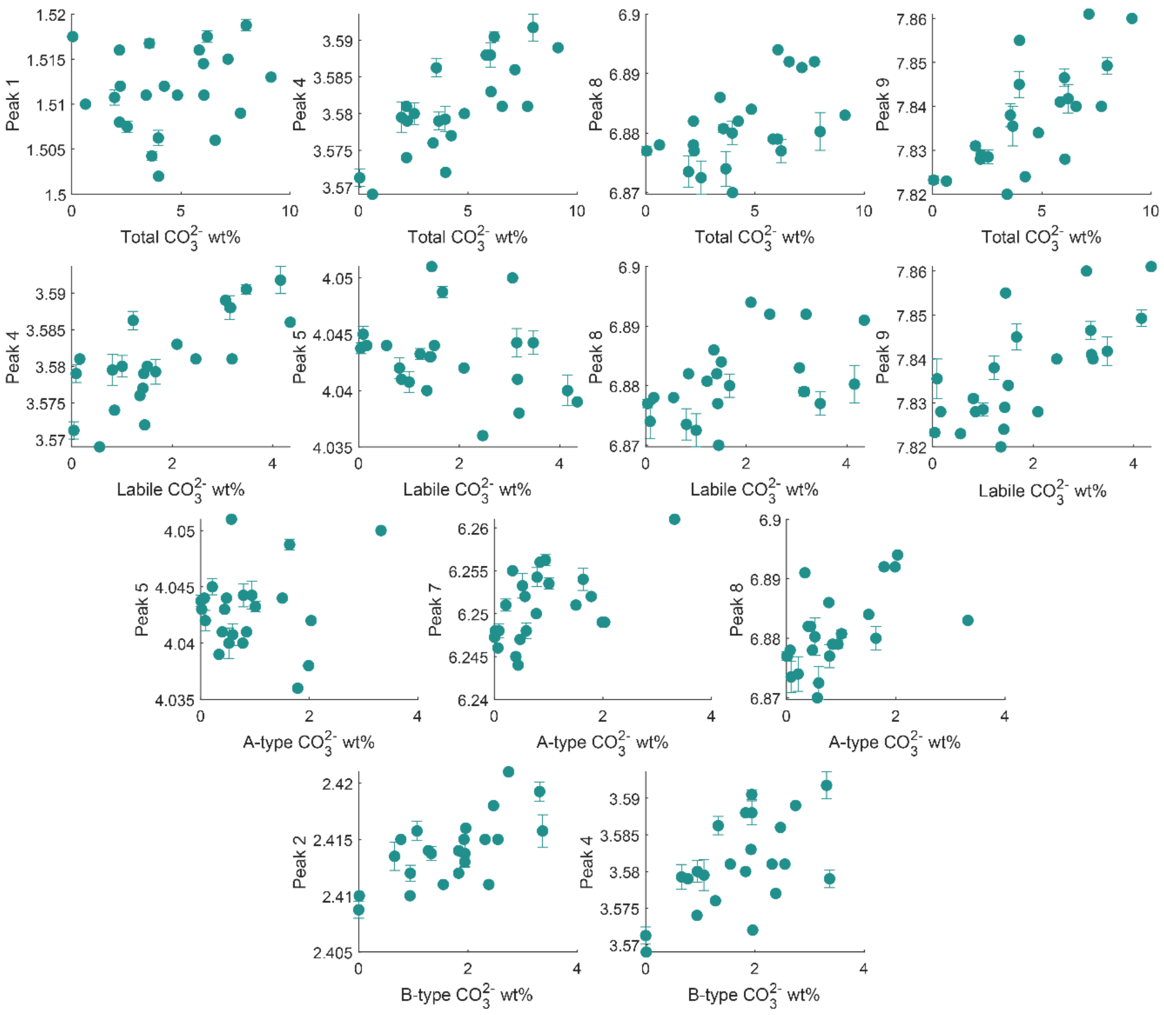

Supplementary Figure S3: Relationships between CO_3^{2-} wt% and peak positions used within stepwise regression models. Error bars represent the standard error of the mean for the peak positions of four repeated scans (present for samples which were repeated, N = 9, and absent for samples which were not repeated, N = 14).

References

1. Kramer, R. Z. *et al.* X-ray crystallographic determination of a collagen-like peptide with the repeating sequence (Pro-Pro-Gly). *J. Mol. Biol.* **280**, 623–638 (1998).
2. Bella, J., Eaton, M., Brodsky, B. & Berman, H. Crystal and molecular structure of a collagen-like peptide at 1.9 A resolution. *Science (80-.).* **266**, 75–81 (1994).
3. Bella, J., Brodsky, B. & Berman, H. M. Hydration structure of a collagen peptide. *Structure* **3**, 893–906 (1995).
4. Bella, J., Liu, J., Kramer, R., Brodsky, B. & Berman, H. M. Conformational Effects of Gly–X–Gly Interruptions in the Collagen Triple Helix. *J. Mol. Biol.* **362**, 298–311 (2006).
5. Vitagliano, L., Berisio, R., Mazzarella, L. & Zagari, A. Structural bases of collagen stabilization induced by proline hydroxylation. *Biopolymers* **58**, 459–464 (2001).
6. Hongo, C. *et al.* Average crystal structure of (Pro-Pro-Gly)9 at 1.0 Å resolution. *Polym. J.* **33**, 812–818 (2001).
7. Berisio, R. & Vitagliano, L. Crystal structure of the collagen triple helix model 3. *Protein Sci.* 262–270 (2002). doi:10.1110/ps.32602.the
8. Emsley, J., Knight, C. G., Farndale, R. W. & Barnes, M. J. Structure of the Integrin α2β1-binding Collagen Peptide. *J. Mol. Biol.* **335**, 1019–1028 (2004).
9. Okuyama, K. *et al.* Crystal structures of collagen model peptides with Pro-Hyp-Gly repeating sequence at 1.26 Å resolution: Implications for proline ring puckering. *Biopolymers* **76**, 367–377 (2004).
10. Kawahara, K. *et al.* Effect of hydration on the stability of the collagen-like triple-helical structure of [4(R)-hydroxyprolyl-4(R)-hydroxyprolylglycine]10. *Biochemistry* **44**, 15812–15822 (2005).
11. Schumacher, M., Mizuno, K. & Bächinger, H. P. The crystal structure of the collagen-like polypeptide (Glycyl-4(R)- hydroxyprolyl-4(R)-hydroxyprolyl)9 at 1.55 Å resolution shows up-puckering of the proline ring in the Xaa position. *J. Biol. Chem.* **280**, 20397–20403 (2005).
12. Hongo, C., Noguchi, K., Okuyama, K., Tanaka, Y. & Nishino, N. Repetitive interactions observed in the crystal structure of a collagen-model peptide, [(Pro-Pro-Gly)9]3. *J. Biochem.* **138**, 135–144 (2005).
13. Okuyama, K. *et al.* High-resolution structures of collagen-like peptides [(Pro-Pro-Gly) 4 -Xaa-Yaa-Gly-(Pro-Pro-Gly) 4]: Implications for triple-helix hydration and Hyp(X) puckering. *Biopolymers* **91**, 361–372 (2009).
14. Okuyama, K. *et al.* Unique side chain conformation of a leu residue in a triple-helical structure. *Biopolymers* **86**, 212–221 (2007).
15. Schumacher, M. A., Mizuno, K. & Bächinger, H. P. The crystal structure of a collagen-like polypeptide with 3(S)-hydroxyproline residues in the Xaa position forms a standard 7/2 collagen triple helix. *J. Biol. Chem.* **281**, 27566–27574 (2006).
16. Okuyama, K. *et al.* Two crystal modifications of (Pro-Pro-Gly) 4 -Hyp-Hyp-Gly-(Pro-Pro-Gly) 4 reveal the puckering preference of Hyp(X) in the Hyp(X):Hyp(Y) and Hyp(X):Pro(Y) stacking pairs in collagen helices. *Acta Crystallogr. Sect. D Biol. Crystallogr.* **66**, 88–96 (2010).
17. Okuyama, K. *et al.* PDB Entry 3A0A: Structure of (PPG)4-OPG-(PPG)4, monoclinic, twinned crystal. (2009). doi:10.2210/pdb3A0A/pdb
18. Okuyama, K. *et al.* Stabilization of triple-helical structures of collagen peptides containing a Hyp-Thr-Gly, Hyp-Val-Gly, or Hyp-Ser-Gly sequence. *Biopolymers* **95**, 628–640 (2011).
19. Okuyama, K. *et al.* Crystal structure of the collagen model peptide (Pro-Pro-Gly) 4 -Hyp-Asp-Gly-(Pro-Pro-Gly) 4 at 1.0 Å resolution. *Biopolymers* **99**, 436–447 (2013).
20. Okuyama, K., Morimoto, T., Hongo, C., Hosaka, N. & Nishino, N. PDB Entry 3AH9: Crystal structure of (Pro-Pro-Gly)9 at 1.1 A resolution. (2010). doi:10.2210/pdb3AH9/pdb
21. Okuyama, K., Miyama, K., Kawajuchi, T. & Nishino, N. PBD Entry 3AI6: Triple-helical structure of (D-Pro-D-Pro-Gly)9 at 1.1 A resolution. (2010).
22. Okuyama, K., Miyama, K., Mizuno, K. & Bächinger, H. P. Crystal structure of (Gly-Pro-Hyp)9: Implications for the collagen molecular model. *Biopolymers* **97**, 607–616 (2012).
23. Shoulders, M. D., Satyshur, K. A., Forest, K. T. & Raines, R. T. Stereoelectronic and steric effects in side chains preorganize a protein main chain. *Proc. Natl. Acad. Sci. U. S. A.* **107**, 559–564 (2010).
24. Byrne, C., McEwan, P. A., Emsley, J., Fischer, P. M. & Cahn, C. End-stapled homo and hetero collagen triple helices: A click chemistry approach. *Chem. Commun.* **47**, 2589–2591 (2011).
25. Gingras, A. R. *et al.* Structural Basis of Mannan-Binding Lectin Recognition by Its Associated Serine Protease MASP-1: Implications for Complement Activation. *Structure* **19**, 1635–1643 (2011).
26. Fallas, J. A., Dong, J., Tao, Y. J. & Hartgerink, J. D. Structural Insights into Charge Pair Interactions in Triple Helical Collagen-like Proteins. *J. Biol. Chem.* **287**, 8039–8047 (2012).
27. Okuyama, K., Haga, M., Noguchi, K. & Tanaka, T. Preferred side-chain conformation of arginine residues in a triple-helical structure. *Biopolymers* **101**, 1000–1009 (2014).
28. Suzuki, H. *et al.* PDB Entry 4OY5: 0.89 Angstrom resolution crystal structure of (Gly-Pro-Hpy)10.

(2015). doi:10.2210/pdb4oy5/pdb
29. Plonska-Brzezinska, M. E. *et al.* Triple helical collagen-like peptide interactions with selected polyphenolic compounds. *RSC Adv.* **5**, 95443–95453 (2015).
30. Xu, T., Zhou, C. Z., Xiao, J. & Liu, J. Unique Conformation in a Natural Interruption Sequence of Type XIX Collagen Revealed by Its High-Resolution Crystal Structure. *Biochemistry* **57**, 1087–1095 (2018).
31. Jalan, A. A. *et al.* Chain alignment of collagen I deciphered using computationally designed heterotrimers. *Nat. Chem. Biol.* **16**, 423–429 (2020).
